# Factors associated with seizure occurrence and long-term seizure control in pediatric brain arteriovenous malformation: a retrospective analysis of 89 patients

**DOI:** 10.1186/s12883-015-0402-5

**Published:** 2015-08-27

**Authors:** Shuang Liu, Hong-xu Chen, Qing Mao, Chao You, Jian-guo Xu

**Affiliations:** Department of Neurosurgery, West China hospital of Sichuan University, 37 Guoxue Street, Chengdu, 610041 People’s Republic of China

## Abstract

**Background:**

Few studies have examined seizures in pediatric brain arteriovenous malformation. In our study, risk factors associated with seizure occurrence and long-term seizure control outcomes after different treatments in pediatric arteriovenous malformation patients were investigated.

**Methods:**

A retrospective analysis was conducted with clinical data from a cohort of 89 pediatric brain arteriovenous malformation patients acquired between 2008 and 2013. Univariate and multivariate analyses were used to assess risk factors associated with seizure incidence. Patients who presented with seizure before treatment were evaluated using the Engel classification during the follow-up period.

**Results:**

A higher risk of seizure occurrence was observed in large size and unruptured brain arteriovenous malformations using multivariate logistic regression analysis (*p* < 0.05). A total of 22 children, who presented with seizure before the interventions, were included in subsequent analysis. During a mean follow-up period of 2.3 years after the intervention, 12 (55 %) of these children were classified as Engel class I after treatment.

**Conclusions:**

Seizures were the most common symptom in unruptured bAVMs. Size of the brain arteriovenous malformation is highly significant to seizure occurrence. Patients with cerebral hemorrhage are prone to having an acute seizure occurrence. The different therapies examined all improved seizure control to varying degrees.

**Electronic supplementary material:**

The online version of this article (doi:10.1186/s12883-015-0402-5) contains supplementary material, which is available to authorized users.

## Background

Brain arteriovenous malformations (bAVMs) are congenital vascular lesions. Patients with bAVMs usually become symptomatic at age 20–40 and typically present with hemorrhage, seizure, or local neurological deficits. Treatment options include microsurgery, radiosurgery, embolization, or a combination of these, with the goal of therapy being complete resection or obliteration of the bAVMs. Treatment reduces the risk of bAVM-related intracranial hemorrhage (ICH) in patients with a history of hemorrhage.

Pediatric bAVMs account for approximately 3–19 % of all bAVMs [1] and are thought to have different clinical, radiological, and outcome characteristics compared to adult bAVMs. Children with bAVMs have a higher annual risk of hemorrhage than adults with bAVMs (2–4 % vs.1–3 % per year) [2], and in around 25 % of these children, hemorrhage is fatal. Because of the high overall lifetime risk of bleeding, an aggressive treatment approach is justified in children with unruptured bAVMs.

Seizures are the second most common initial manifestation in children with bAVMs and can affect their intellectual capacity and neuropsychological status, among other deleterious effects [3]. Though prevention of bleeding rather than seizure control is the primary reason for considering surgery in pediatric patients with unruptured bAVMs, seizure occurrence and its post-operative outcome deserve consideration. Seizure control rate is low in patients with unruptured bAVMs [4]. Seizures can present as a complication following bAVM surgery. Additionally, surgery can raise the risk of seizure in patients with ruptured bAVMs [5–8].

The current literature has primarily focused on bAVM-related hemorrhage or seizure in adult cohorts. So far, little is known about the incidence and outcomes of seizure in pediatric bAVMs. In this study, we summarize our experience with the treatment of a serial cohort of 89 pediatric bAVMs. We focus on their long-term seizure control outcomes after different treatments and identify the factors associated with pre-therapy seizure incidence.

## Methods

### Patient population

**Fig. 1:**
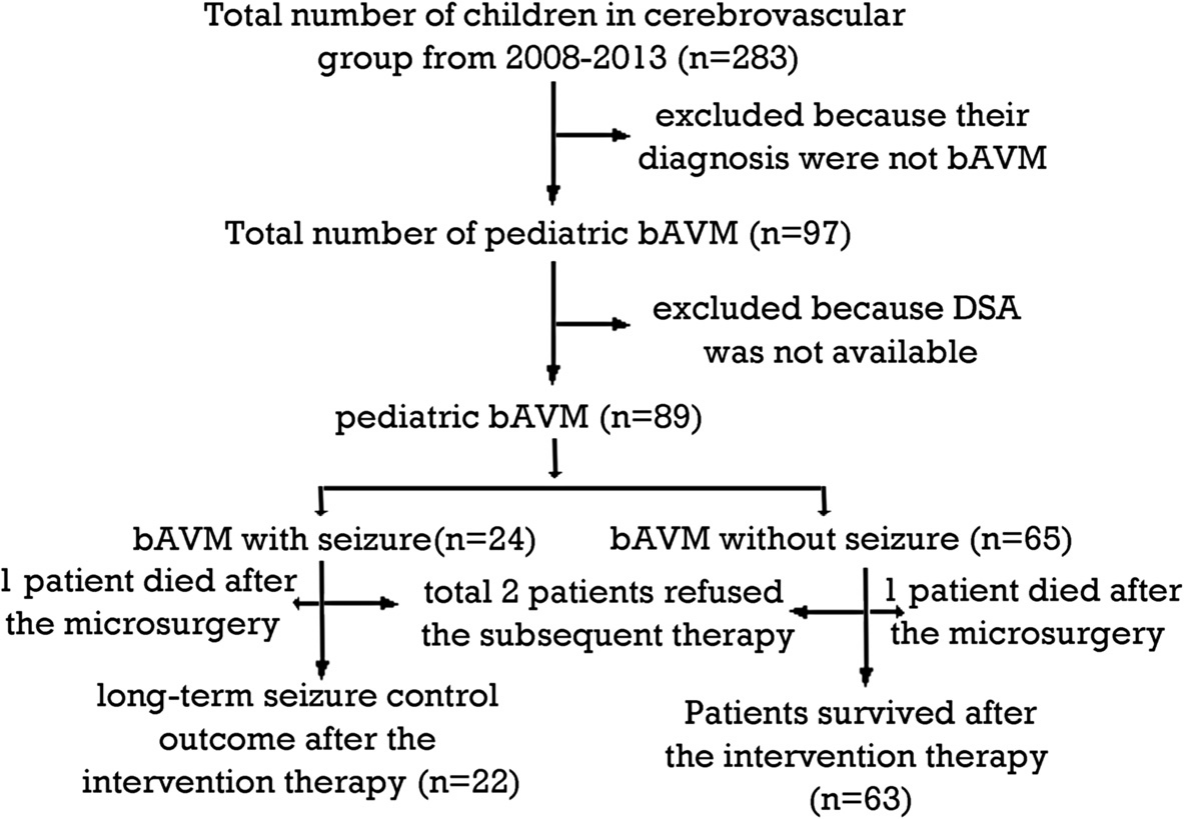
Distribution of the patients selected in our study

We retrospectively reviewed all children aged up to 18 years who were admitted with cerebrovascular diseases to our department of neurosurgery at the West China hospital in China between March 2008 and December 2013. The inclusion criteria were defined as ruptured or unruptured intracranial AVM diagnosed by digital subtraction angiography before the intervention therapy. We excluded cavernous malformation, dural and pial arteriovenous fistulas, and vein malformations. Eighty-nine children were identified consecutively (Fig. 1). The following information was collected during review of the medical records: age, sex, clinical presentations, neurological signs at admission, history of intracranial hemorrhage, hemorrhage broken into the ventricle (IVH), the DSA finding, the treatment modality, seizure type, duration of seizure, seizure frequency, and seizure outcome after treatment. History of intracranial hemorrhage was defined as the patient having presented with hemorrhage in their patient history. The DSA finding included the nidus location, size, involvement of the eloquent area, and the Spetzler-Martin grade [9].

Clinical outcome was assessed via the regular outpatient and telephone interview. We also made a special follow-up telephone call to identify each child’s recent status. In all the patients for whom we received follow-up information, DSA/CT angiography was generally performed 1–3 months after the microsurgery or the embolization. Adjuvant therapy was sometimes performed when a patient presented with a residual lesion.

### Seizure assessment

During long-term follow-up, seizure outcomes were determined using the modified Engel Classification scale [10]. Class I was defined as seizure free, auras only, or seizures upon drug withdrawal only; Class II as a 90 % reduction in seizure frequency or a single post-operative seizure; Class III as a 50 % reduction in seizure frequency or no change in seizure frequency; and Class IV as an increase in postoperative seizure frequency. Patients were defined as having new-onset seizure when they developed epilepsy after their bAVMs treatment, during the follow-up period. These new-onset seizure patients were excluded from further analysis of seizure control outcome. We defined the duration of seizure as time between first seizure onset and the treatment.

### Treatment modalities

Pediatric bAVMs cases were referred to our department from other hospitals in the Sichuan province (population of 80 million). Except in cases requiring emergency ICH evacuation, for small bAVMs (size < 3 cm) or intermediate bAVMs (size ≥ 3 cm and size ≤ 6 cm), we sought to excise the nidus through open microsurgery. For large bAVMs (size > 6 cm), we performed endovascular treatment followed by partial or complete obliteration of the nidus to achieve flow reduction and partial nidus obliteration. When complete obliteration was unlikely, we performed additional stereotactic radiosurgery or microsurgery. In our center, microsurgery was usually considered the first-choice treatment and Stealth intraoperative neuronavigation was usually used to assist localization. However, radiosurgery was considered in some cases in which bAVMs were difficult to access surgically or in which operative risk was high. All radiosurgery patients were treated with the Leksell Gamma Knife model C (Elekta, Sweden) using existing planning software.

When a patient underwent multimodality AVM treatment, we classified them in the microsurgery group if they underwent microsurgery, in the embolization group if they did not undergo microsurgery (irrespective of whether they received stereotactic radiosurgery or not), and in the radiosurgery group if they only underwent this therapy.

Patients with a history of seizure generally received antiepileptic drugs (AEDs) before the therapy. During the microsurgery and embolization, sodium valproate was administered intravenously and tapered post-operatively over 3 days, after which oral AEDs were administered. The oral outpatient AEDs most commonly used was levetiracetam or oxcarbazepine, which were administered according to the child’s weight. Patients were tapered off AEDs when they remained seizure-free for 3 months after the intervention. Patients or their relatives gave informed consent for participation in the study, as well as for the publication of their clinical details. The ethical approval for this study, including retrospective data collection, was granted by the Biological and Medical Ethics Committee of West China Hospital.

### Statistical analysis

We analyzed children’s sex, age, admission Glasgow coma scale (GCS) score, ICH, IVH, bAVM size, venous drainage, eloquent area, location, and Spetzler-Martin grade to determine whether these factors were associated with preoperative seizure occurrence. The chi-squared test was used for categorical variables (e.g., sex) and Student’s *t*-test was used for continuous variables (e.g., age). For further analysis of preoperative factors associated with seizures, significant factors from univariate analyses were entered into a multivariate logistic regression. All statistical analyses were 2-tailed (α = 0.05), and a *p*-value of ≤0.05 was considered statistically significant. Statistical analyses were performed using SPSS version 21.0 (IBM Corporation, Armonk, NY, USA).

## Results

### Study population

The study group consisted of 89 children of whom 52 were boys (58.4 %) and 37 were girls (41.6 %). The mean age was 12.2 years (range 1–18 years). In these 89 patients, 2 patients refused the subsequent intervention therapy in our hospital when they finished their DSA examinations. For the remaining 87 patients, mean follow-up duration was 1.8 years (ranging from immediately following treatment to 68 months). 3 patients eventually discontinued follow-up treatment after having undergone it for at least 7 months.

According to the Glasgow outcome scale (GOS), a good recovery was achieved in 82 out of the remaining 87 patients (95.4 %) and only 3 patients were moderately disabled (3.4 %). The mortality rate was 2.3 % (*n* = 2). These two patients underwent open-operation therapy to evacuate the hemorrhage and AVM nidus. One patient death was related to the pre-operative deep comatose state of the patient, while the other patient died of brain infection and hydrocephalus after the surgery.

Among the patients, 45 (50.6 %) presented with headache, 24 (27.0 %) with seizures, 12 (13.5 %) with neurological deficits, and 3 (3.4 %) were asymptomatic. 67 (75.3 %) children sustained intracranial hemorrhage due to bAVMs. The initial clinical manifestations when they were admitted to the hospital included abrupt headache (*n* = 38), disturbance of consciousness (*n* = 13), focal neurological deficits (*n* = 10), seizure (*n* = 5) and dizziness (*n* = 1). Of the 3 asymptomatic patients, 1was found to present with bAVMs in an MRI scan that was being conducted because of short stature, 1 was found after a minor car accident, and 1 had fever and headache and was diagnosed with viral encephalitis. For the latter patient, the lesion was found during a follow-up brain CT examination. The patient’s clinical presentation disappeared when his encephalitis was cured. The clinical and angioarchitectural features of the 89 patients are summarized in Table 1.

**Table 1:** Factors associated with seizure occurrence in the 89 pediatric brain arteriovenous malformation patients

Factors	Group	No seizure	Seizure	*P* value	
				Univariate	Multivariate
Sex	Male	35	17	0.149	
	Female	30	7		
Age	Mean ± SD	12.11 ± 3.554	12.25 ± 3.814	0.870	
Admission GCS score	≤8	11	4	0.837	
	9–12	5	1		
	≥13	49	19		
ICH	Yes	56	11	0.000	0.007
	No	9	13		
IVH	Yes	24	5	0.151	
	No	41	19		
Size	Small	43	4	0.000	0.005
	Medium	16	14		
	Large	6	6		
Venous drainage	Deep	40	8	0.030	0.305
	Superficial	22	12		
	Mixed	3	4		
Eloquent area	Yes	23	6	0.354	
	No	42	18		
Location	Frontal	8	5	0.083	
	Temporal	14	3		
	Occipital	6	3		
	Parietal	6	6		
	Multilobar	5	5		
	Cerebellum	6	0		
	Callosum	4	1		
	IVH	2	0		
	Thalamus/basal Ganglia	14	1		
Spetzler-Martin grade	Grade 1	13	1	0.090	
	Grade 2	21	10		
	Grade 3	22	7		
	Grade 4	8	3		
	Grade 5	1	3		

*ICH* Intracerebral hemorrhage, *GCS* Glasgow coma scale, *IVH* Hemorrhage broken into the ventricle

During the follow-up period, 5 (5.7 %) of the 87 patients exhibited rebleeding. Of these, 3 had previously undergone only radiosurgery as an initial therapy. After the recurrent hemorrhage, one of these three patients underwent microsurgery and subsequent radiosurgery therapy, while the other two patients underwent embolization and radiosurgery therapy. Their last postoperative angiograms showed obliteration rates of 90 % (patient 1 in Table 2), 80 and 90 %, respectively. Of the other 2 patients that exhibited rebleeding, one had undergone an initial therapy of embolization and subsequent radiosurgery, while the other patient had undergone an initial therapy of only embolization. After rebleeding, the two patients underwent microsurgery and embolization along with radiosurgery, respectively. Their latest DSA examinations showed no residual AVMs.

**Table 2:** Characteristics and seizure therapy of the 4 new-onset seizure patients

No.	Therapy	Seizures appearing	Type of seizure	AED drugs	Seizure frequency	Number of seizures	Duration of seizure	Obliteration rate
1	Microsurgery	3th days	CP	levetiracetam	2 in the first month	2	1 month	DSA- after the surgery
2	Embolization	<24 hours	GTC	lamotrigine and levetiracetam	monthly	11	10 months	DSA-
3	Embolization	<24 hours	SP	oxcarbazepine	3 in 1 year	10	3 years	80 %
4	Radiosurgery	7th days	CP	levetiracetam	4 in 1 year	10	3 years	90 %

*GTC* Generalized tonicoclonic seizure, *ABS* Absence seizure, *SP* Simple seizure, *CP* Complex seizure, *DSA-* Digital substract angiography negative

### Factors associated with seizure occurrence

In the univariate analysis, factors not significantly associated with seizure included sex (*χ*^2^ = 2.082, *p* = 0.149), age (*p* = 0.870), admission GCS score (*χ*^2^ = 0.357, *p* = 0.837), IVH (*χ*^2^ = 2.066, *p* = 0.151), eloquent area (*χ*^2^ = 0.860, *p* = 0.354), location (*χ*^2^ = 13.949, *p* = 0.083) and Spetzler-Martin grade (*χ*^2^ = 8.039, *p* = 0.090). There were 3 features significantly associated with initial seizure presentation: size (*χ*^2^ = 17.273, *p* = 0.000), hemorrhage (*χ*^2^ = 15.313, *p* = 0.000), and venous drainage (*χ*^2^ = 7.019, *p* = 0.030). After multivariate analysis, only larger size (OR = 3.063, 95 % CI = 1.414–6.634, *p* = 0.005) and hemorrhage (OR = 0.196, 95 % CI = 0.060–0.640, *p* = 0.007) remained significantly associated with initial seizure presentation (Table 1).

### Hemorrhage and seizure outcome

There were 67 (75.3 %) patients that presented with hemorrhage, 29 of who presented with hemorrhage broken into the ventricle. 15 patients with intracerebral hemorrhage (22.4 %) reported acute symptomatic seizures. Of these, 11 (16.4 %) had seizures before the intervention therapy, and 10 were included in further analysis of seizure outcome. In the long-term follow-up, 6 of these 10 patients achieved Engel class I, 2 patients achieved Engel class II, and 2 patients achieved Engel class III. Neither of the 2 class III patients showed complete obliteration of bAVMs at the last imaging examination of the follow-up. In contrast, among the 22 unruptured bAVMs patients, as many as 13 presented with seizure and of these 13, 12 were included in further analysis of seizure outcomes. In the long-term follow-up, 6 of these patients were Engel class I and the other 6 were Engel class II.

A total of 4 patients developed new-onset seizure after the therapy and all of them presented with hemorrhage. Of these, 2 patients had undergone embolization therapy, while the remaining 2 were treated with microsurgery and radiosurgery, respectively (Table 2). The median number of days to seizure post-therapy was 3 (range 1–7). Neither of the two embolization patients had seizures before treatment, but both had seizures immediately post-therapy (<24 h). One of the embolization patients received a second embolization and subsequent radiosurgery 6 months after the initial embolization therapy. His seizures disappeared 4 months after the second therapy. The other embolization patient received a second embolization and AED therapy (patient 3 in Table 2), but still experienced occasional seizures. The seizures ceased after the patient underwent radiosurgery approximately 3 years later, while the complete obliteration of the nidus was still not achieved at that time (patient 4 in Table 2).

### Seizure type/duration and seizure outcome

There were a total of 24 patients who had seizures occur before therapy. One patient who refused subsequent intervention therapy and one patient who died after the surgery were excluded from further analysis of seizure outcome. Of the remaining 22 children with seizure, the mean follow-up time was 2.3 years (range 12–60 months). There were 12 (55 %) Engel Class I patients, 8 (36 %) Engel Class II patients, and 2 (9 %) Engel Class III patients. Most children experienced generalized tonicoclonic seizures (64 %), whereas others experienced absence (4 %), simple partial (18 %), or complex partial (14 %) seizures. Engel class I was achieved by 3 of 4 (75 %) patients that presented simple seizures, 1 (100 %) patient that presented absence seizure and 8 of 14 (57 %) patients that presented generalized tonic-clonic seizures. All 3 patients that presented complex seizures were classified as Engel class II.

Of the patients who had seizures before therapy, 8 of them had presented seizures for longer than 6 months. 2 patients still had monthly seizure occurrences upon receiving combined AEDs therapy before the intervention. After intervention therapy, the DSA examinations for both patients were negative for the presence of bAVMs. However, both patients still presented seizures and were classified as Engel Class II. 10 of 14 (71 %) patients with seizure duration of less than 6 months achieved Engel Class I, while only 2 of 8 (25 %) patients with seizure duration longer than 6 months achieved Class I (Additional file 1: Table S1). 5 of the 8 patients with longer seizure durations had negative DSA results.

### Therapy modality and seizure outcome

Nine of 22 patients were treated with microsurgery, 3 patients with embolization, and 1 patient with radiosurgery. In addition, 2 patients underwent embolization prior to AVM resection, 1 patient underwent microsurgery and subsequent radiosurgery, and 6 patients underwent combined embolization and radiosurgery. The mean follow-up time in embolization group (*n* = 10) and microsurgery group (*n* = 11) were 23.4 and 28.8 months, respectively. Six of 10 (60 %) patients in the embolization group and 5 of 11 (45 %) patients in the microsurgery group achieved Engel class I. The patient who underwent radiosurgery alone was seizure free without requiring AEDs at follow-up. By the last follow-up, 13 patients had achieved complete obliteration of the nidus. Among them, 8 patients achieved Engel class I, and the remaining 5 patients achieved Engel class II.

## Discussion

Seizure is the second most common symptom of bAVMs following hemorrhage, with up to 40 % of bAVMs patients presenting with seizures [11, 12]. Even when the bAVMs nidus is obliterated or removed through microsurgery, radiosurgery, or embolization, many patients still suffer from seizures that can affect their quality of life [5, 8, 12, 13]. In our study, out of 89 children with bAVMs, 67 (75.3 %) had hemorrhaged and 24 (27.0 %) had experienced seizures before therapy. In the long-term follow-up to these seizure patients, 12 (55 %) achieved an Engel class I status after the therapy, with a mean follow-up period of 2.3 years.

The mechanisms by which bAVMs lead to epileptogenesis remain unclear, although many studies have identified significant risk factors for epilepsy [7, 14, 15]. Hol et al. studied clinical predictors of seizure incidence and seizure outcomes in bAVMs patients with seizure and observed that male sex, age less than 65 years, bAVMs size greater than 3 cm, and bAVMs location in the temporal lobe are associated with pretreatment seizure [16]. Shankar et al. analyzed angioarchitectural characteristics of unruptured bAVMs in a consecutive series of patients presenting with seizures and proposed that venous outflow stenosis, long pial draining vein and location of bAVMs are the strongest predictors of seizures [17]. A recent prospective study investigating seizures as the initial presentation of bAVMs found that predisposing factors include male sex, increasing bAVMs size, superficial venous drainage, and location in the frontal lobe and arterial border-zone [18].

In our study, we found that only size and hemorrhage were significant factors associated with seizure occurrence in pediatric bAVMs, while age, gender, and other factors were unrelated to seizure occurrence. Previous studies have shown an association between larger bAVMs sizes and greater seizure incidence [8, 17, 19]. bAVM size may affect the occurrence of seizures by creating a hypoxic environment in surrounding brain tissue, a condition known to lead to seizures. Larger bAVMs tended to have more arteriovenous shunting of blood, a factor associated with focal cerebral ischemia [17].

We found that hemorrhage is another significant factor correlated with seizure, consistent with findings from a number of previous studies. The products of blood metabolism, such as hemosiderin, can cause focal cerebral irritation, which may lead to seizure. This has been shown in animal models of focal epilepsy produced by iron deposition on the brain cortex [20]. However, in our study, as many as 13 of 22 patients with unruptured bAVMs (i.e., without bleeding) presented with seizures. The finding that seizure occurrence in unruptured bAVMs is significantly higher than that in the hemorrhage group (59 % vs.16 %) is somewhat paradoxical given that we found hemorrhage is a significant factor correlated with seizure. A plausible interpretation is that many bAVMs are not detected unless they become symptomatic e.g., when they present with seizures or when they rupture. As such, in unruptured bAVMs initially identified because of seizures, prompt therapy is taken to avoid hemorrhage [21]. Consistent with this, seizures were the most common symptom in unruptured bAVMs. In a previous report, seizure risk was highest during the first 24 h after hemorrhagic stroke and tended to decrease thereafter [20]. The majority of our patients developed acute symptomatic seizures in the early stage of hemorrhage, before therapy. This finding supports an epileptogenic role of blood evacuation. However, it is worth noting that patients with new-onset seizure after the therapy all had a history of hemorrhage. Seizure occurrence in cortical regions may be a complication of invasive microsurgery. Moreover, the residual hemorrhage, the partial obliteration of lesion, and the sudden change in hemodynamic upon embolism therapy may also contribute to seizure onset. In the long-term follow-up, the non-hemorrhage group had a slightly better seizure control rate compared to the hemorrhage group. However, considering the limited patient number in the study, it is difficult to draw any definitive conclusion on the influence of hemorrhage on long-term seizure control outcome. It remains unclear whether evacuation of the hemorrhage could lead to better seizure control than embolization and radiosurgery.

It has been suggested that pre-operative seizure type and duration are prognostic factors for post-operative seizure control. Hol et al. found that shorter seizure duration and generalized tonicoclonic seizure type are associated with good seizure outcomes [16]. Our data showed 14 of 22 pediatric patients had generalized tonicoclonic seizures and 8 of them (57 %) achieved Engel Class I after the therapy. This seizure control rate was lower than that for patients with simple seizures, but better than that for patients with complex seizures. In our series, the longer the duration of seizure, the more likely the preoperative seizure was to belong term repetitive and refractory. Only 25 % of our pediatric patients with seizure histories longer than 6 months were seizure-free post-operatively, but up to 71 % of patients with shorter seizure histories were seizure-free. To achieve a more favorable seizure outcome after surgery for those patients with long duration and frequent seizures, we advise a more aggressive surgical resection, including the excision of cortical lining of AVMs, which can exhibit hemosiderin deposits, gliosis, neuronal degradation, or demyelization [3, 22].

Recently, Baranoski et al. compared seizure control rates upon treatment of bAVMs with different therapeutic modalities and concluded that microsurgical resection of bAVMs led to higher rates of seizure-free patients and better seizure control than radiosurgery or embolization [6, 23]. This may be attributable to the presence of an epileptogenic focus at the site of the bAVMs, as suggested by the presence of gliosis and hemosiderin borders, which are considered epileptogenic [4, 21].

There have been few studies about the relationship between seizure and embolization of bAVMs in adults or children. One possible reason for this may be that embolization therapy was once primarily used as a conjunction therapy with microsurgery or radiosurgery [7, 8]. Of the 30 patients in a study by Lv X et al., 4 patients that underwent complete embolization of their bAVMs achieved excellent seizure control [8]. In our study, though the mean obliteration rate was 90 %, 60 % of patients that underwent embolization therapy achieved Engel class I. However, both of the embolization patients had new-onset seizures immediately after the procedure. Kede los also reported that following embolization with the liquid embolic agent Onyx, seizure occurred in 45 % (*n* = 9) of 20 patients and that the median time to seizure post-Onyx was 7 days (range 0.3–210). The development of edema and inflammation surrounding the nidus following Onyx administration may play a role, especially in patients with partial obliteration of bAVMs [7, 22].

Radiosurgery can eventually achieve a higher post-operative seizure-free rate than microsurgery when the lesion is completely obliterated [6, 24]. However, 2–3 years may be required to fully obliterate the lesion, and patients risk the possibility of hemorrhage. Meanwhile, some studies report that radiosurgery can affect epileptogenesis from tissue surrounding the bAVMs in a manner independent of radiation-induced thrombosis of bAVMs, and can reduce seizures even before complete obliteration of bAVMs [16]. This is further evidenced by our study, as one of the new-onset seizure patients who underwent radiosurgery had seizures cease before the nidus was fully obliterated.

The decision to surgically treat potentially epileptogenic unruptured bAVMs is still a subject of controversy. Even with AED therapy, seizure response remains variable for these patients. Some people become drug resistant or undergo refractory seizures and need an intervention therapy in order to control their seizures. Most studies have reported that microsurgery has a good seizure control rate and can decrease the rate of spontaneous hemorrhage [23, 24]. However, in a recent prospective, population-based observational study, Josephson et al. found no significant difference in the 5-year risk of seizure in adults between the conservative and treatment groups, irrespective of whether the bAVMs had presented with hemorrhage or epileptic seizures [4]. One possible explanation for these results is that their patients who underwent treatment for bAVMs may have had a higher baseline propensity for seizures, and that their seizures may have been refractory to conservative care, thus resulting in bAVMs treatment [23, 24]. In our serial cohort, patients with unruptured bAVMs have had varying degrees of improvement in control of their seizures. Seizure-free states were achieved in 60 % of patients in the embolization group, 45 % of those in the surgery group, and 100 % of those in the radiosurgery group. Given that children have long life spans, seizure can greatly impact their life and bring unexpected risk. We suggest active intervention treatment to reduce the risk of hemorrhage and improve seizure control, especially for medical management of refractory seizures. For some post-therapy refractory seizures, we would consider epilepsy surgery to settle the seizure or to remove the epileptic focus. Individual treatment strategies should be made based on the presentation and angio-architectural characteristics of the bAVM to determine the optimal treatment modality.

Our study has several limitations. First, our study was a retrospective analysis, and therefore, was subject to biases inherent to that study design. Bias in the assessment of patient seizures and in selection of patients for different therapeutic modalities certainly influenced our results. Second, the number of patients may not be large enough to draw definitive conclusions regarding treatment modality and the influence of hemorrhage on long-term seizure control. Additionally, study of the relationship between obliteration rate and seizure control outcome requires a large sample size and a prospective trial. Future work is required to reach a more definitive conclusion.

## Conclusion

Our study demonstrates seizure remains the most common symptom in unruptured bAVMs pediatric patients and most children present with generalized seizures. Size of the brain arteriovenous malformation is highly significant to seizure occurrence. Patients with cerebral hemorrhage are prone to having an acute seizure occurrence. Radiosurgery, embolization, and microsurgery could all influence seizure control and reduce the risk of rebleeding in pediatric bAVMs patients. What the best therapy for seizure control in unruptured bAVMs is remains a subject of debate. Moreover, considering the young age at presentation, a careful follow-up is needed to prevent future bAVMs and seizure recurrence in pediatric bAVMs patients.

## Additional file

Additional file 1: Table S1. Characteristics and seizure outcomes of the 22 pediatric brain arteriovenous malformation patients. EVE, endovascular embolization; MS, microsurgery; SRS, stereotactic radiosurgery; NA, not clear. 18*: This patient underwent the combination of EVE and SRS as an initial therapy and postoperative angiograms showed obliteration rates of about 60 %. When he exhibited rebleeding, MS therapy was taken, and post-operation DSA examination showed no residual bAVM. (DOCX 18 kb)

## Abbreviations

ICH: Intracerebral hemorrhage
bAVMs: Brain arteriovenous malformations
DSA: Digital subtraction angiography
GCS: Glasgow coma scale
AEDs: Antiepileptic drugs

## References

[1] Nair AP, Kumar R, Mehrotra A, Srivastava AK, Sahu RN, Nair P (2012). Clinical, radiological profile and outcome in pediatric Spetzler-Martin grades I-III arteriovenous malformations. Childs Nerv Syst.

[2] Rubin D, Santillan A, Greenfield JP, Souweidane M, Riina HA (2010). Surgical management of pediatric cerebral arteriovenous malformations. Childs Nerv Syst.

[3] Scott RC (2014). What are the effects of prolonged seizures in the brain?. Epileptic Disord.

[4] Josephson CB, Bhattacharya JJ, Counsell CE, Papanastassiou V, Ritchie V, Roberts R, Sellar R, Warlow CP, Al-Shahi Salman R (2012). Seizure risk with AVM treatment or conservative management: prospective, population-based study. Neurology.

[5] Hyun SJ, Kong DS, Lee JI, Kim JS, Hong SC (2012). Cerebral arteriovenous malformations and seizures: differential impact on the time to seizure-free state according to the treatment modalities. Acta Neurochir (Wien).

[6] Baranoski JF, Grant RA, Hirsch LJ, Visintainer P, Gerrard JL, Günel M, Matouk CC, Spencer DD, Bulsara KR (2013). Seizure control for intracranial arteriovenous malformations is directly related to treatment modality: a meta-analysis. J NeurointervSurg.

[7] de Los RK, Patel A, Doshi A, Egorova N, Panov F, Bederson JB, Frontera JA (2011). Seizures after Onyx embolization for the treatment of cerebral arteriovenous malformation. Interv Neuroradiol.

[8] Lv X, Li Y, Jiiang C, Yang X, Wu Z (2010). Brain arteriovenous malformations and endovascular treatment: effect on seizures. Interv Neuroradiol.

[9] Spetzler RF, Martin NA (1986). A proposed grading system for arteriovenous malformations. J Neurosurg.

[10] Engel J, Van Ness PC, Rasmussen TB, Engel J (1993). Outcome with respect to epileptic seizures. Surgical treatment of the epilepsies.

[11] Hofmeister C, Stapf C, Hartmann A, Sciacca RR, Mansmann U, terBrugge K, Lasjaunias P, Mohr JP, Mast H, Meisel J (2000). Demographic, morphological, and clinical characteristics of 1289 patients with brain arteriovenous malformation. Stroke.

[12] Turjman F, Massoud TF, Sayre JW, Viñuela F, Guglielmi G, Duckwiler G (1995). Epilepsy associated with cerebral arteriovenous malformations: a multivariate analysis of angioarchitectural characteristics. AJNR Am J Neuroradiol.

[13] Thorpe ML, Cordato DJ, Morgan MK, Herkes GK (2000). Postoperative seizure outcome in a series of 114 patients with supratentorial arteriovenous malformations. J Clin Neurosci.

[14] Englot DJ, Young WL, Han SJ, McCulloch CE, Chang EF, Lawton MT (2012). Seizure predictors and control after microsurgical resection of supratentorial arteriovenous malformations in 440 patients. Neurosurgery.

[15] Galletti F, Costa C, Cupini LM, Eusebi P, Hamam M, Caputo N, Siliquini S, Conti C, Moschini E, Lunardi P, Carletti S, Calabresi P (2014). Brain arteriovenous malformations and seizures: an Italian study. J Neurol Neurosurg Psychiatry.

[16] Hoh BL, Chapman PH, Loeffler JS, Carter BS, Ogilvy CS (2002). Results of multimodality treatment for 141 patients with brain arteriovenous malformations and seizures: factors associated with seizure incidence and seizure outcomes. Neurosurgery.

[17] Shankar JJ, Menezes RJ, Pohlmann-Eden B, Wallace C, terBrugge K, Krings T (2013). Angioarchitecture of brain AVM determines the presentation with seizures: proposed scoring system. AJNR Am J Neuroradiol.

[18] Garcin B, Houdart E, Porcher R, Manchon E, Saint-Maurice JP, Bresson D, Stapf C (2012). Epileptic seizures at initial presentation in patients with brain arteriovenous malformation. Neurology.

[19] Sturiale CL, Rigante L, Puca A, Di Lella G, Albanese A, Marchese E, Di Rocco C, Maira G, Colicchio G (2013). Angioarchitectural features of brain arteriovenous malformations associated with seizures: a single center retrospective series. Eur J Neurol.

[20] Beghi E1, D’Alessandro R, Beretta S, Consoli D, Crespi V, Delaj L, Gandolfo C, Greco G, La Neve A, Manfredi M, Mattana F, Musolino R, Provinciali L, Santangelo M, Specchio LM, Zaccara G (2011). Incidence and predictors of acute symptomatic seizures after stroke. Neurology.

[21] Josephson CB, Leach JP, Duncan R, Roberts RC, Counsell CE, Al-Shahi Salman R (2011). Seizure risk from cavernous or arteriovenous malformations: prospective population-based study. Neurology.

[22] Fierstra J, Conklin J, Krings T, Slessarev M, Han JS, Fisher JA, Terbrugge K, Wallace MC, Tymianski M, Mikulis DJ (2011). Impaired peri-nidal cerebrovascular reserve in seizure patients with brain arteriovenous malformations. Brain.

[23] Wang JY, Yang W, Ye X, Rigamonti D, Coon AL, Tamargo RJ, Huang J (2013). Impact on seizure control of surgical resection or radiosurgery for cerebral arteriovenous malformations. Neurosurgery.

[24] Chen CJ, Chivukula S, Ding D, Starke RM, Lee CC, Yen CP, Xu Z, Sheehan JP (2014). Seizure outcomes following radiosurgery for cerebral arteriovenous malformations. Neurosurg Focus.

