# Supplementary material for: Factors associated with seizure occurrence and long-term seizure control in pediatric brain arteriovenous malformation: a retrospective analysis of 89 patients
Source: BMC Neurol. 2015 Aug 27;15:155. doi: 10.1186/s12883-015-0402-5 (PMC4550043; doi:10.1186/s12883-015-0402-5)
Supplement: Additional file 1: Table S1. — Characteristics and seizure outcomes of the 22 pediatric brain arteriovenous malformation patients. EVE, endovascular embolization; MS, microsurgery; SRS, stereotactic radiosurgery; NA, not clear. 18*: This patient underwent the combination of EVE and SRS as an initial therapy and postoperative angiograms showed obliteration rates of about 60 %. When he exhibited rebleeding, MS therapy was taken, and post-operation DSA examination showed no residual bAVM. (DOCX 18 kb) [file 12883_2015_402_MOESM1_ESM.docx]

**Additional file 1: Table S1. Characteristics and seizure outcomes of the 22 pediatric brain arteriovenous malformation patients**

EVE, endovascular embolization; MS, microsurgery; SRS, stereotactic radiosurgery; NA, not clear

18*: This patient underwent the combination of EVE and SRS as an initial therapy and postoperative angiograms showed obliteration rates of about 60%. When he exhibited rebleeding, MS therapy was taken, and post-operation DSA examination showed no residual bAVM.

| NO | Sex | Age | Seizure  type | Seizure  duration | Frequency | Numbers of seizure | Bleeding | Grade | Therapy | Obliteration rate | Engel class | Follow-up time( months) |
| --- | --- | --- | --- | --- | --- | --- | --- | --- | --- | --- | --- | --- |
| 1 | f | 8 | GTC | 2months | 1 in 2 months | 1 | yes | 3 | EVE | DSA- | Ⅰ | 15 |
| 2 | m | 11 | GTC | 14days | 1 in 14 days | 1 | yes | 5 | EVE+SRS | 85 | Ⅰ | 18 |
| 3 | m | 14 | ABS | 12months | 2 in 6 months | 4 | no | 1 | SRS | NA | Ⅰ | 20 |
| 4 | m | 13 | GTC | 12months | 3 in 6 months | 6 | no | 3 | EVE | 95 | Ⅱ | 14 |
| 5 | f | 16 | CP | 1day | 3 in 1 day | 3 | yes | 2 | MS | DSA- | Ⅱ | 12 |
| 6 | m | 8 | CP | 1day | 2 in 1 day | 2 | yes | 2 | MS | NA | Ⅱ | 12 |
| 7 | m | 12 | GTC | 10months | monthly | 10 | no | 2 | MS | DSA- | Ⅱ | 43 |
| 8 | f | 10 | GTC | 3days | 1 in 3 days | 1 | no | 2 | MS | DSA- | Ⅰ | 36 |
| 9 | m | 15 | GTC | 12hours | 2 in 12 hours | 2 | Yes | 3 | MS | DSA- | Ⅰ | 46 |
| 10 | f | 1 | GTC | 1month | 1 in 1 month | 1 | No | 3 | MS | DSA- | Ⅰ | 42 |
| 11 | m | 11 | GTC | 2days | 2 in 2 days | 2 | Yes | 3 | SRS, 2years later hemorrhage, underwent MS + SRS | 90 | Ⅲ | 36 |
| 12 | m | 11 | GTC | 14days | 2 in 14 days | 2 | yes | 3 | EVE+SRS | 85 | Ⅲ | 21 |
| 13 | m | 11 | CP | 10months | 1 in 2 months | 5 | no | 2 | MS | DSA- | Ⅱ | 19 |
| 14 | m | 16 | GTC | 12months | monthly | 12 | no | 4 | EVE+MS | DSA- | Ⅱ | 17 |
| 15 | m | 10 | SP | 15hours | 2 in 15 hours | 2 | yes | 2 | EVE+ SRS | DSA- | Ⅰ | 20 |
| 16 | m | 15 | GTC | 12days | 3 in 12 days | 3 | yes | 2 | MS | DSA- | Ⅰ | 32 |
| 17 | m | 17 | SP | 10years | 1 in 4 years | 3 | no | 4 | EVE | 90 | Ⅱ | 26 |
| 18^*^ | m | 13 | SP | 4months | 1 in 4 months | 1 | no | 5 | EVE+SRS ,2years later hemorrhage, underwent MS | DSA- | Ⅰ | 58 |
| 19 | f | 9 | GTC | 10days | 3 in 10 days | 3 | yes | 5 | EVE + SRS | 90 | Ⅰ | 13 |
| 20 | f | 16 | GTC | 12years | 1 in 2 years | 6 | no | 2 | EVE + MS | DSA- | Ⅱ | 25 |
| 21 | f | 14 | SP | 2years | 1 in 6 months | 4 | no | 2 | MS | DSA- | Ⅰ | 22 |
| 22 | m | 9 | GTC | 1month | 2 in 1 month | 2 | no | 2 | EVE + SRS | NA | Ⅰ | 24 |
